# The detrimental effects of glucocorticoids exposure during pregnancy on offspring’s cardiac functions mediated by hypermethylation of bone morphogenetic protein-4

**DOI:** 10.1038/s41419-018-0841-1

**Published:** 2018-08-06

**Authors:** Jieying Peng, Yuhao Zhou, Zhiyu Zhang, Zhiming Wang, Lingtong Gao, Xiao Zhang, Zhou Fang, Guangyao Li, Huaiyan Chen, Hongxing Yang, Lu Gao

**Affiliations:** 1 Department of Physiology, College of Basic Medical Sciences, Second Military Medical University, Shanghai, 200433 China; 2 Department of Health Management, Changzheng Hospital, Second Military Medical University, Shanghai, 200003 China; 3 Chenshan Plant Science Research Center, Chinese Academy of Sciences, Shanghai Chenshan Botanical Garden, Shanghai, 201602 China

## Abstract

The intra-uterine and external environmental factors not only affect the early development of fetuses, their interaction with genesis will also substantially program the physiological functions of offspring throughout life. Synthetic glucocorticoid (GC) is widely used for the management of women at risk of preterm birth or undergone autoimmune diseases. However, excess GC might cause a number of chronic diseases in later life. In the present study, we set up a programming rat model by daily injection of dexamethasone (DEX) since 14.5 dpc until labor, and found that the cardiac functions were significantly compromised in the male offspring compared with that exposed to NS, especially after ischemia/reperfusion (I/R), due to the increased infarction and apoptosis of myocardium. Using MeDIP sequencing, we identified four genes involved in the cardiac muscle cell differentiation and development pathway exhibited increased methylation in their promoter regions, among which, bone morphogenetic protein-4 (BMP4) expression is coordinately decreased in myocardium from male mice prenatally exposed to DEX. The programming effect of DEX on cardiomyocytes apoptosis was found to be dependent on mitochondria dysfunction, whereas the breakdown of mitochondrial membrane potential (ΔΨm) and the decrease of ATP production from mitochondria caused by prenatal DEX exposure both can be restored by BMP4 predisposing on neonatal cardiomyocytes 24 h prior to I/R. Inversely consistent with ΔΨm and ATP production, the release of reactive oxygen species was dramatically elevated in cardiomyocytes, which was significantly inhibited in the presence of BMP4 prior to I/R. These findings suggested that the excess GC exposure during pregnancy increases the susceptibility of male offspring’s heart to “second strike”, due to the decrease of BMP4 expression caused by the hypermethylation on *Bmp4* promoter and the absence of BMP4 protective effect in cardiomyocytes, making the addition of BMP4 a promising treatment for the congenital heart disease under such circumstances.

## Introduction

The intra-uterine and external environmental factors not only affect the early development of fetuses, their interaction with genesis will also substantially program the physiological functions of offspring throughout life^1^. Since Barkers et al. found the association of adverse intra-uterine environment with an increased risk of hypertension and ischemic heart disease in adulthood^2^, a large number of findings in many species have demonstrated that neurological, endocrine, metabolic and cardiovascular function and dysfunction in adulthood have developmental origins^3–5^. The maternal chronic hypoxia during late gestation could increase the cardiac vulnerability to ischemia and reperfusion injury in adult offspring in rats via downregulating the expression of PKC{epsilon} (protein kinase C epsilon) in myocardium^6^. Fetal undernutrition in utero by maternal food restriction significantly increases angiotensinogen and endothelin-1 expression in the left ventricles of adult offspring, which subsequently causes the increase in systolic blood pressure, perivascular fibrosis of the coronary artery, cardiac cardiomegaly and cardiomyocyte enlargement^7^.

Endogenous glucocorticoids (GCs) provide a critical developmental trigger. In most mammalian species, a surge in levels of GCs in the fetal circulation occurs during late gestation. This surge is essential for normal development and maturation of many organs, including thyroid, kidney, brain, pituitary and the fetal lung^5,8^. Our recent study found that the upregulation of lysophosphatidylcholine acyltransferase-1 by GCs in fetal lungs near term not only promotes the production of pulmonary surfactant, but also increases the production of platelet-activating factor, which consequently facilitates the initiation of labor^9^. Due to its important roles in promoting fetal organ maturation, GCs are widely used for the management of women at risk of preterm birth during late gestation. Meanwhile, GCs are also first-line treatment for the fetuses with congenital adrenal hyperplasia^10^ or the pregnant women with asthma^11^ or an autoimmune disease (such as idiopathic thrombocytopenic purpura and systemic lupus erythematosus)^12,13^.

However, this developmental trigger must be applied at a precise stage of maturation with precise dosage, as prenatal exposure to excess GCs (either exogenous or endogenous) might compromise the development of various organ systems, and cause a number of chronic diseases in later life, including diabetes mellitus, obesity, psychiatric diseases and cardiovascular disease^3,5^. Most studies related to the adverse effects of GC focused on its roles in regulating hypothalamic–pituitary–adrenal axis^5^, whereas its effects on specific organs were largely ignored. Regarding cardiovascular diseases, prenatal GC exposure-induced adult hypertension is intensively studied, and the mechanisms have been discovered as activation of renin–angiotensin system and the increased expression of angiotensin receptors, Agtr1 and Agtr2^14–16^. Although epidemiological study showed that the low birth weight caused by prenatal GC exposure significantly increases the incidence of coronary heart disease and ischemic cardiomyopathy^17^, the mechanisms underlying this remain largely unknown.

In this study, we found that prenatal GC exposure could increase the susceptibility of myocardium to I/R injury in adult offspring, uncovered the methylation profile of gene promoters in adult myocardium after prenatal GC exposure and the target genes that may be involved into the programming effects of GCs.

## Materials and methods

### Animal model

**Fig. 1:**
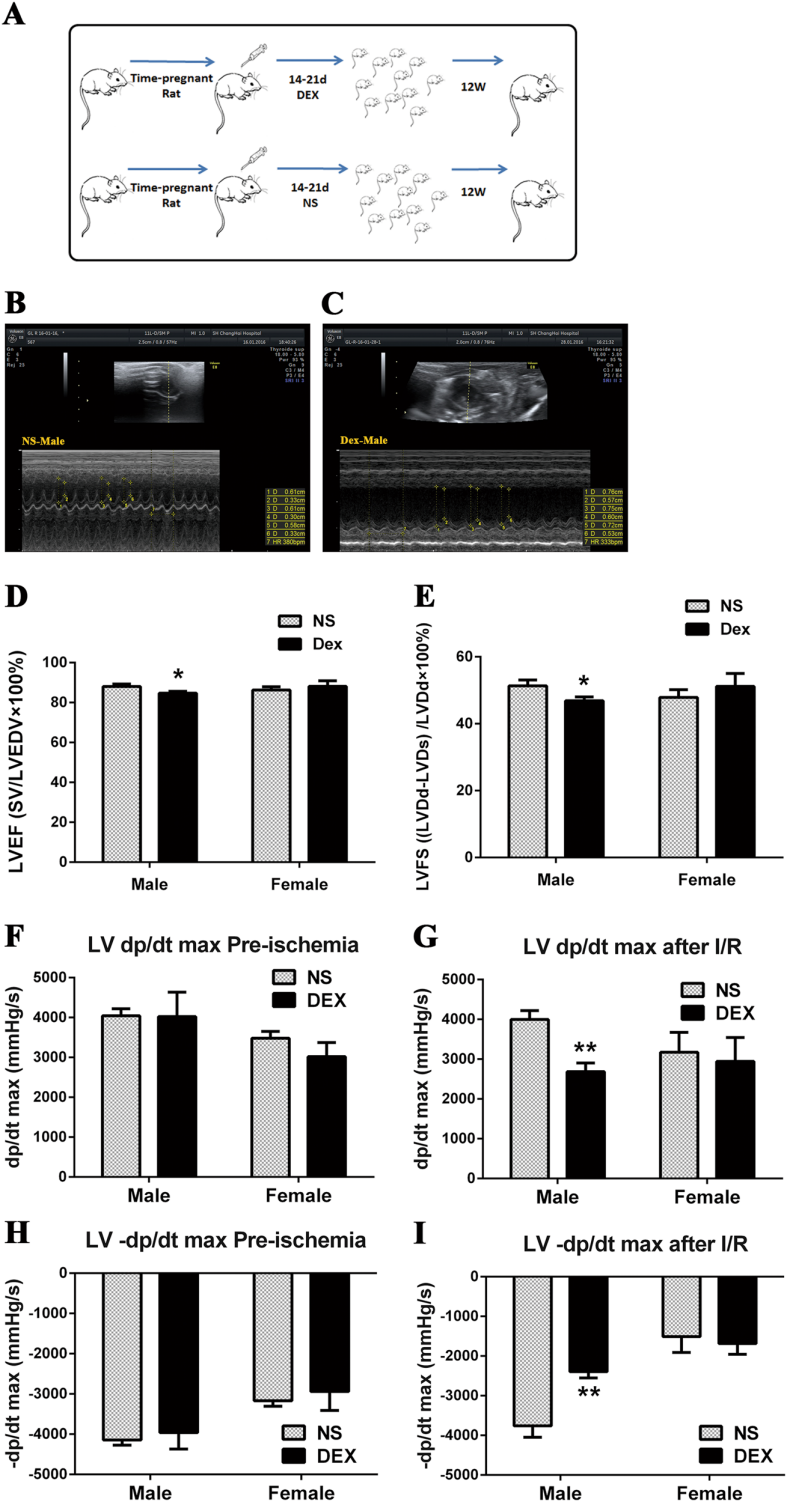
Cardiac functions were compromised in prenatally DEX-exposed adult male offspring. **a** Diagram for the animal model procedure. Timed-pregnant mice at 14.5 dpc were injected subcutaneously with DEX (0.1 mg/kg/d) or NS at the same volume once a day until labor. Representative echocardiographic images of the adult male offspring prenatally exposed to NS (**b**) and DEX (**c**). The LVEF (**d**) and LVFS (**e**) were slightly but significantly decreased in the male offspring exposed to DEX during late gestation compared with that exposed to NS, which is NOT evident in female offspring. The LV dp/dt max and LV –dp/dt max in adult offspring did not show significant differences between NS group and DEX group before ischemia (**f**, **h**). The LV dp/dt max and LV –dp/dt max both significantly decreased after ischemia/reperfusion (I/R) injury in adult male offspring but not in adult female offspring, both prenatally exposed to DEX (**g**, **i**). NS, normal saline; DEX dexamethasone, LVEF left ventricular ejection fraction, LVFS left ventricular fractional shortening, dp/dt max maximal rate of left ventricle systolic pressure change, –dp/dt max maximal rate of left ventricle diastolic pressure change. Data shown are mean ± SEM. *p < 0.05, **p < 0.01

Adult Sprague-Dawley rats were housed under pathogen-free conditions, maintained on a 12-h dark/12-h light cycle, and allowed free access to a standard pellet chow and water. Male and female were mated according to the estrus cycle of rats at 1800 h and separated the following morning at 0600 h. Pregnancy was indicated by the presence of a vaginal plug next morning, which was designated as 0.5 days post coitus (dpc). The time to labor was documented upon delivery of the first pup or by the presence of a litter. A total of 20 pregnant rats were randomly divided into dexamethasone (DEX) group and normal saline (NS) group. The pregnant rats in DEX group were subcutaneously injected with DEX (0.1 mg/kg/d) since 14.5 dpc until labor; the rats in NS group accepted the same volume of NS injection. The newborn pups were kept with their mothers until weaning (Fig. 1a). After weaning, the offspring were maintained in the same environment until grown-up (12 weeks and 24 weeks).

### Echocardiography

The adult offspring rats were lightly anesthetized with intraperitoneal (i.p.) injection of 3% pentobarbital sodium and scanned by two-dimensional M-mode echocardiography (GE Vivid 7) with a 75 MHz cardiac probe to record physiological data. Measurement techniques were consistent with the American Society of Echocardiography conventions^18^. Briefly, the probe was placed on the left side of sternum with 30-degree angle to sternal midline, showing the left ventricular long axis section; then the probe was rotated 90 degrees clockwise to show the left ventricular short axis section. Specifically, the left ventricular end-diastolic anterior wall thickness, posterior wall thickness, the left ventricular end-diastolic dimension (LVEDD), the left ventricular end-systolic dimension (LVESD) and heart rate were measured and recorded. Left ventricular fractional shortening (LVFS), an index of LV systolic function, was calculated by this equation: LVFS = (LVEDD – LVESD)/LVEDD × 100%. Left ventricular end-diastolic volume (LVEDV) = 1.04 × LVEDD^3^; left ventricular end-systolic volume (LVESV) = 1.04 × LVESD^3^. Left ventricular ejection fraction (LVEF) = (LVEDV – LVESV)/LVEDV × 100%. All ultrasound measurements were taken on an average of five cardiac cycles, and all investigators performing echocardiographic acquisition or analysis were blinded to treatment group.

### The in vivo ischemia/reperfusion (I/R) model

Rats were anesthetized with 3% pentobarbital sodium i.p. and ventilated with a rodent ventilator (CWE Incorporated, USA). Myocardial ischemia and reperfusion was conducted as previously described^19^. Briefly, a parasternal incision was made by cutting the left third and fourth ribs and intercostal muscles with surgical scissors. Myocardial ischemia was induced by passing a 6-0 silk suture beneath the left anterior descending artery at a point 1–2 mm inferior to the left auricle. The suture was tightened over a piece of PE-20 tubing (Becton, Dickinson, USA) for 30 min and then released for 2 h.

### Cardiac functions measured by arterial catheter

Before or after I/R, the rat was incised from the midline of the neck, and the subcutaneous tissue and muscles were blunt separated. The right common carotid artery was dissociated for 1–2 cm, with 2–3 silk sutures underneath. The distal end of common carotid artery was ligated, and the proximal end was clamped with arterial clip. A “V”-shaped incision was cut using ophthalmic scissors between the distal end of the ligation line and the arterial clip. Insert the arterial catheter filled with heparin into the proximal end of common carotid artery. Connect the arterial catheter with the physiological parameter recorder, then open the arterial clamp and slowly push the catheter into the left ventricle. Left ventricular systolic pressure, left ventricular diastolic pressure, left ventricular end-diastolic pressure, maximal rate of the increase of left ventricular pressure (+dp/dt max) and maximal rate of the decrease of left ventricular pressure (−dp/dt max) were recorded.

### Determination of infarct size and the area at risk

After I/R, the suture was tightened again, and rats were intravenously injected with Evans blue (Sigma-Aldrich, St. Louis, MO). When the skin of chest wall became blue, the heart was immediately excised and quick-frozen in liquid nitrogen. Then, the heart was cut into 2-mm thick slices parallel to the atrioventricular groove and were immerged into 10% paraformaldehyde in phosphate-buffered saline (PBS; pH 7.4) for 2 h. The heart sections not stained with Evans blue were identified as the risk area. The slices were then incubated with 1% 2,3,5-triphenyltetrazolium chloride (TTC; Amresco, Solon, OH) in phosphate buffer (pH 7.4) at 37 °C for 20 min to identify the infarct area. TTC was catalyzed by dehydrogenase enzymes to formazan, which stains viable myocardium with dark red. The infarct area that does not contain dehydrogenase enzymes is not able to convert TTC into formazan and thus remains pale in color^19,20^. The area at risk and the infarct area were quantified using Image J (https://imagej.nih.gov/ij/). Infarct size was expressed as a ratio of the infarct area and the area at risk. Apoptotic cardiomyocytes in the border zone of the infarct area were detected by TdT-mediated dUTP nick-end labeling (TUNEL) staining.

### TUNEL of the infarct left ventricular tissue

After I/R and Evans blue staining, the left ventricular heart sections were fixed with 10% paraformaldehyde and embedded into paraffin. Paraffin sections were cut into 5 mm thick slides, rehydrated, and incubated in proteinase K buffer at 37 °C for 30 min to retrieve antigens. The cell membrane was permeabilized with Triton X-100. Apoptotic cardiomyocytes were detected with TUNEL staining using an in situ cell death detection kit (Roche), according to the manufacturer’s instructions. To determine the number of nuclei, the slides were incubated with 4,6-diamidino-2-phenylindole (DAPI) for 10 min at 37 °C in dark and rinsed with PBS (pH 7.4) three times, with 5 min for each rinse. Then, the slides were mounted with antifade mounting medium. The TUNEL signals were observed with a fluorescence microscopy (NIKON ECLIPSE TI-SR). In each heart section, five slides with an interval of > 10 mm were examined. Ten fields were randomly selected in each slide, and the cells positive with TUNEL staining (green signals) and DAPI staining (blue signals) were counted by an observer blinded with the experimental groups (magnifications × 200). Apoptosis was determined as a ratio of the number of TUNEL-positive cells to total nuclear number in each field.

### Detection of global DNA methylation status in offspring myocardium

The genomic DNA of offspring myocardium was extracted using tissue genomic DNA extraction kit (Biotek, Beijing). The purity of the DNA was checked spectroscopically on the basis of the ratio between OD260 and OD280, and the concentration was determined by measuring absorbance at OD260. The genome-wide methylation was detected using the MethylFlash™ Methylated DNA Quantification Kit (Epigentek Group Inc., Farmingdale, NY), according to the manufacture’s instruction. Briefly, 100 ng of the DNA sample was bound to strip wells that are specifically treated to have a high DNA affinity. The methylated fraction of DNA is detected using capture and detection antibodies and then quantified colorimetrically by reading the absorbance at the wavelength of 450 nm in a microplate spectrophotometer. The amount of methylated DNA is proportional to the OD (optical density) intensity measured.

### Real-time PCR

Total RNA from tissues or cells was extracted using an RNeasy Mini Kit (Qiagen). The purity and integrity of the RNA were checked spectroscopically and by gel electrophoresis before use. Quantification of total RNA was performed by measuring absorbance at OD260. RNA was treated with DNase (Invitrogen) to remove any contaminating DNA, and 2 μg were reversed transcribed using the SuperScript III cDNA First-Strand Synthesis Kit (Invitrogen). The specificity of the primers was verified by examining the melting curve. Product purity was confirmed by gel electrophoresis. For quantitative analysis of mRNA, a CFX Connect Real-Time PCR Detection System (Bio-Rad) was employed using SYBR Green Real-time PCR Master Mix (TOYOBO) for the detection of PCR products. The cycling conditions were 95 °C for 3 min, followed by 40 cycles of 95 °C for 15 s, and 60 °C for 30 s. As a negative control for all of the reactions, distilled water was used in place of complementary DNA. Amplification of the housekeeping gene GAPDH was determined for sample loading and normalization. Each sample was amplified in triplicate, and the mean of Ct value was calculated. The relative fold changes were calculated using the comparative Ct method (2^–ΔΔCt^)^21^.

### Western blot

Total protein extracts were prepared from tissues and cells using RIPA buffer (Cell Signaling). Equivalent amounts of protein determined by BCA assay were resolved by 10% Bis-Tris gel electrophoresis and blotted to Immobilon PVDF membranes (Millipore). The following primary antibodies and dilutions were used: anti-DNMT1 (1:1000; ab13537, Abcam); anti-DNMT3a (1:1000; ab188470, Abcam); anti-DNMT3b (1:1000; ab79822, Abcam); anti-DNMT3L (1:1000; ab3493, Abcam); anti-BMP4 (1:1000; ab39973, Abcam); anti-TBX3 (1:1000; ab154828, Abcam); anti-ACADM (1:1000; ab92461, Abcam); anti-caspase 3 (1:1000; 9662S, Cell Signaling); anti-GAPDH (1:5000; 5174S, Cell Signaling). Horseradish peroxidase-conjugated anti-rabbit or anti-mouse IgG (Proteintech) was used as secondary antibody. The membranes were developed using Supersignal West Pico Chemiluminescent substrate (Thermo Scientific). To normalize sample loading and transfer, the ratio of band intensities to GAPDH was obtained to quantify the relative protein expression level.

### MeDIP sequencing

MeDIP sequencing was performed by KangChen Bio-tech, Shanghai, China. Briefly, DNA samples were fragmented to a size range of ~200–1000 bp with a Diagenode Bioruptor (Diagenode, Denville, NJ, USA). About 1 μg of fragmented DNA was ligated to Illumina’s genomic adapters with Genomic DNA Sample Kit (#FC-102–1002, Illumina), following the manufacturer’s instructions. Around 300–900 bp ligated DNA fragments were further immunoprecipitated by anti-5-methylcytosine antibody (Diagenode). The enriched DNA was amplified by PCR and purified by agarose gel. The completed libraries were quantified by Agilent 2100 Bioanalyzer. The libraries were denatured with 0.1 M NaOH to generate single-stranded DNA molecules, captured on Illumina flow cell, amplified in situ. The libraries were then sequenced on the Illumina HiSeq 2000 using TruSeq Rapid SBS Kit (#FC-402–4001, Illumina).

After sequencing images generated, the stages of image analysis and base calling were performed using Off-Line Basecaller software (OLB V1.8). After passing Solexa CHASTITY quality filter, the clean reads were aligned to Rattus_norvegicus genome (UCSC RN5) using BOWTIE software (V2.1.0). Aligned reads were used for peak calling, both mRNA and LncRNA-associated MeDIP-enriched regions (peaks) with statistically significant were identified for each sample, using a q-value threshold of 10^−5^ by MACS v2. Both mRNA and LncRNA-associated MeDIP-enriched regions (peaks) were annotated by the nearest gene using the newest UCSC RefSeq database. Both mRNA and LncRNA associated differentially methylated regions (DMRs) within promoter between two groups with statistical significance were identified by diffReps (cut-off: log2FC = 1.0, *p*-value = 10^−4^). Both mRNA and LncRNA-associated DMRs within promoter were annotated by the nearest gene using the UCSC RefSeq and database of multiple databases integration.

### Gene ontology analysis

To investigate the significance of the altered methylation observed by MeDIP-seq, we analyzed genes that exhibited greater than a 2-fold change (log2) in methylation (DEX group vs. NS group) using Gene Ontology (GO) analysis (http://www.geneontology.org). The ontology covers three domains: biological process, cellular component and molecular function. Fisher’s exact test is used to find if there is more overlap between the differentially expressed (DE) list and the GO annotation list than would be expected by chance. The *p*-value denotes the significance of GO terms enrichment in the DE genes. The lower the *p*-value, the more significant the GO term (*p*-value ≤ 0.05 is recommended).

### The neonatal cardiomyocytes culture and in vitro hypoxia/reoxygenation model

Primary cultures of neonatal cardiomyocytes were prepared according to the methods previously described^19^. Briefly, cardiac cells were dissociated from 1-day-old rat pups from NS group or DEX group with 0.1% trypsin, 0.025% type II collagenase and 0.01% DNase. To selectively enrich myocytes, dissociated cells were plated and kept at 37 °C with 5% CO2 for 1 h. The non-myocytes attached readily to the bottom of the culture dish. The resultant suspension of cardiomyocytes were plated into culture plates at a density of 5 × 10^5^ cells/ml and cultured in humidified 5% CO_2_, 95% air at 37 °C. 5-Bromo-2′-deoxyuridine (100 μM) was added during the first 48 h to prevent proliferation of non-myocytes. All experiments were performed using cells cultured for day 4–day 5, when the spontaneous cell beating was observed.

To establish the hypoxia/reoxygenation model, the culture medium was changed into Dulbecco’s modified Eagle’s medium without glucose and serum, and the cells were exposed to hypoxia (95%N_2_/5% CO_2_) for 4 h, followed by reoxygenation for 2 h. The cells were pretreated with BMP4 (50 ng/ml) for 24 h before the hypoxia/reoxygenation procedure. The control group was cultured under normal air condition and treated with vehicle.

### Assessment of apoptotic cell death in cultured cardiomyocytes

Apoptotic death of cardiomyocytes was determined by TUNEL. TUNEL was performed with a cell apoptosis detection kit (Roche). Briefly, after the cells were fixed and permeabilized with 0.1% Triton X-100, the cell slides were incubated with 50μl TUNEL reaction mixture in humidified atmosphere for 60 min at 37 °C in the dark. To detect the nuclei, the cells were incubated with DAPI for 10 min at room temperature in the dark, rinsed with PBS three times with 5 min for each rinse, and then observed under a fluorescence microscopy. Cell apoptosis was determined as the ratio between the number of TUNEL-positive nuclei and that of DAPI-positive nuclei from five randomly selected fields (magnification × 400).

### Flow cytometry

The apoptosis assay of cardiomyocytes was conducted using eBioscience™ Annexin V Apoptosis Detection Kit (Invitrogen) following the manufacturer’s protocols. Samples were detected by flow cytometry (FACSVerse; BD Biosciences) and analyzed with the FACSSuite software (BD Biosciences). Fragments of dead cells and debris were gated out by forward- and side-scatter analysis.

### Assessment of mitochondrial membrane potential

Cardiomyocyte mitochondrial damage was assessed by using the mitochondrial membrane potential assay kit with JC-1, according to the protocols provided by the manufacturer (Beyotime, Hangzhou, China). JC-1 is a marker of mitochondrial activity, which is most widely applied for detecting mitochondrial depolarization occurring in the early stages of apoptosis. JC-1 aggregates in the mitochondria and gives off a red fluorescence in healthy cells. In apoptotic cells, JC-1 fails to aggregate in the mitochondria as a result of altered mitochondrial transmembrane potential and remains in the cytoplasm in its monomer form, which exhibits green fluorescence.

Briefly, the cells were incubated with 0.5 ml JC-1 staining solution for 20 min at 37 °C, and the fluorescent signals were observed by a fluorescence microscopy and measured by a CYTATION3 multifunctional microplate reader (Biotek). The red fluorescent signals were excited at 525 nm and detected at 590 nm, and the green fluorescence was excited at 490 nm and detected at 530 nm. The data were shown as the ratio of red signals versus green signals.

### Cellular ATP level assessment

Intracellular ATP levels were assessed through the firefly luciferase-based ATP assay kit (Beyotime), according to manufacturer’s instruction. Briefly, the cultured neonatal cardiomyocytes were lysated using ATP lysis buffer. After centrifugation to remove cell debris, 20 μl of supernatant was added to 100 μl of ATP detection solution at its working dilution. Luminance (RLU) was measured using a CYTATION3 multifunctional microplate reader (Biotek) with an integration time of 10 s per well and the concentration of ATP was calculated according to the standard curve. ATP levels were normalized to the protein levels.

### Measurement of mitochondrial superoxide

Mitochondria-mediated reactive oxygen species (ROS) generation was detected with the mitochondrial superoxide indicator MitoSOX-Red (Invitrogen). Briefly, the cells were washed twice in PBS and incubated with 5 mol/l of MitoSOX-Red working solution for 10 min at 37 °C, protected from light. Then, the cells were washed gently three times with warm buffer, and the fluorescent signals were measured using a CYTATION3 multifunctional microplate reader (Biotek) with the excitation wavelength at 510 nm and emission wavelength at 580 nm, or analyzed using FACS Verse flow cytometer (BD).

### Statistics

All data are presented as mean ± SEM or as the mean percentage of control ± SEM, in some cases. Differences between groups were analyzed by one-way analysis of variance followed by a Dunnett’s multiple comparisons test. The *p*-values smaller than 0.05 were considered to be statistically significant.

## Results

### Cardiac functions were compromised in prenatally GC-exposed offspring

When the offspring grew adult and reached 12 weeks, we determined the cardiac functions using echocardiography. It was found that the LVEF and LVFS of male offspring exposed to DEX during late gestation were significantly decreased, which was not observed in female offspring (Figs. 1b-e). To further confirm the changes of cardiac functions, we determined the maximal rate of left ventricle systolic pressure change ( + dp/dt max) and the maximal rate of left ventricle diastolic pressure change (–dp/dt max) of adult offspring using heart floating catheter, and no significant differences were shown between NS group and DEX group (Figs. 1f, h). However, after I/R injury of myocardium, we found the ± dp/dt max were both significantly decreased in adult male rats but not in female rats from DEX group, compared with those from NS group (Figs. 1g, i).

In the elder offspring, which were 24 weeks old, ultrasonic cardiogram showed that the LVEF and LVFS of male and female offspring exposed to DEX during late gestation did not manifest significant changes compared with those exposed to NS (Supplemental Fig. 1a,b). However, the ± dp/dt max were both significantly decreased in adult male offspring from DEX group after I/R, but not in female offspring (Supplemental Fig. 1C and D), which is similar to the phenomenon we observed in 12-week-old offspring.

### Prenatal GC exposure increases myocardium infarct size and cardiomyocytes apoptosis in vivo

**Fig. 2:**
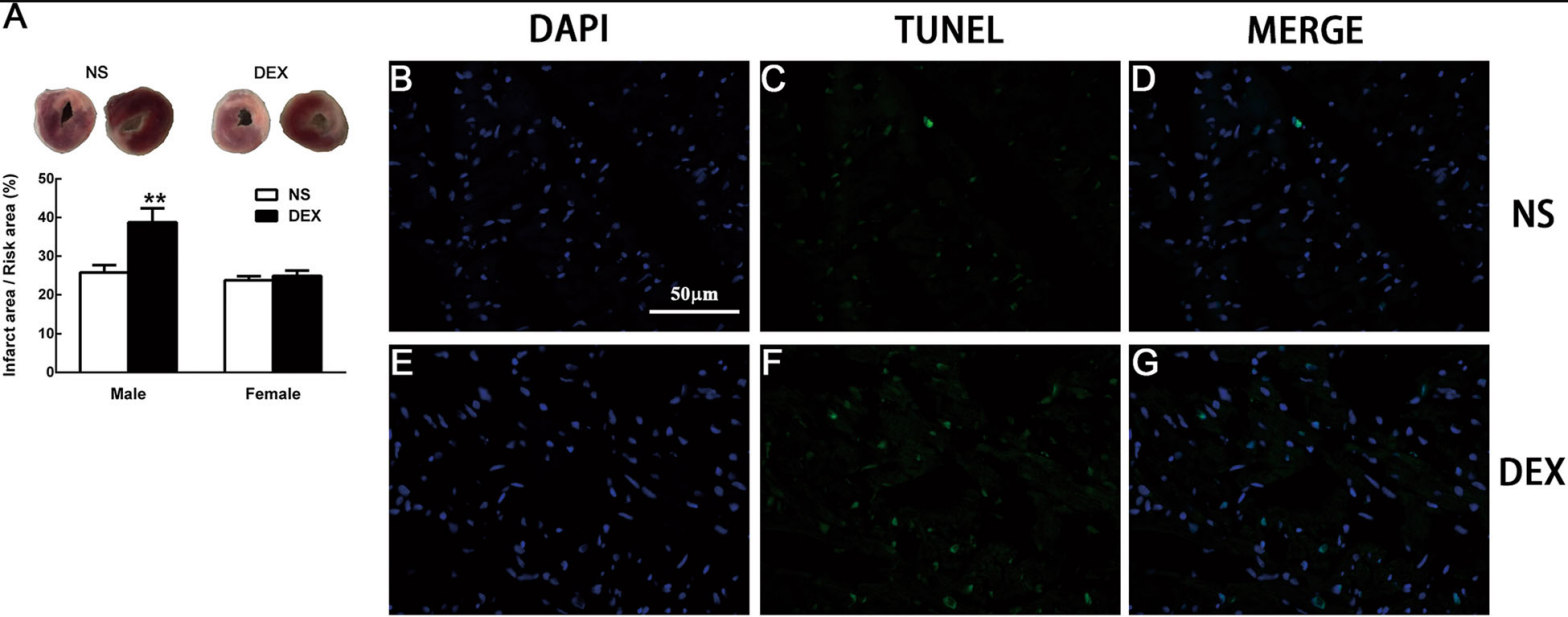
Prenatal GC exposure increased offspring’s myocardium infarct size and cardiomyocytes apoptosis after I/R in vivo. The TTC staining showed that myocardium infarct size was observably increased in prenatally DEX-exposed adult male offspring than that exposed to NS (**a**); TUNEL staining was performed on myocardium undergoing I/R. Although TUNEL-positive cells were rarely detected in NS group (**b**–**d**), DEX exposure led to much more extensive cardiomyocytes apoptosis (**e**-**g**). Nucleus stained with DAPI (blue)

As shown in Fig. 2a, myocardium infarct size was significantly increased in the male offspring exposed to DEX during pregnancy than that exposed to NS, indicated by the TTC staining. The number of apoptotic cells was significantly increased in prenatally DEX-exposed rats compared with that in NS-exposed control (Figs. 2b–g), suggesting that the increased susceptibility of cardiomyocytes to injury (I/R) programmed by prenatal DEX exposure is the major reason that contributes to the compromise of cardiac functions.

### DNA methyltransferases and global genomic DNA methylation profile in myocardium

**Fig. 3:**
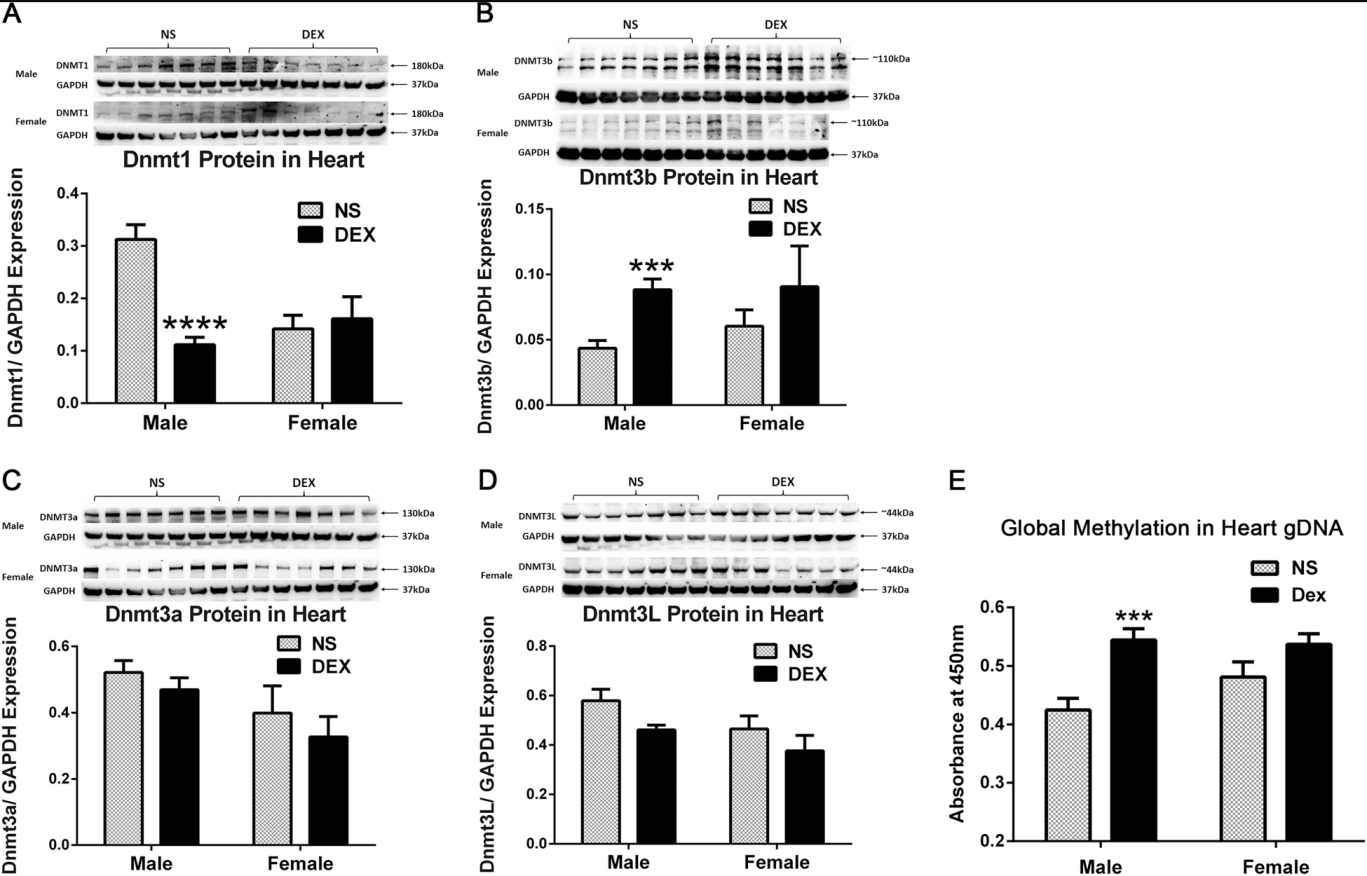
DNA methyltransferases expression and global genomic DNA methylation profile in myocardium tissues. DNMT1 (**a**), DNMT3b (**b**), DNMT3a (**c**) and DNMT3L (**d**) expression was shown in the myocardium tissues of prenatally DEX-exposed offspring and prenatally NS-exposed offspring. Representative immunoblots were shown in the upper panel, corresponding to each densitometric scan normalized to GAPDH as an internal standard, which were shown in the lower panel. Global DNA methylation increased in DEX-exposed adult male offspring’s myocardium compared with that from NS-exposed adult male offspring (**e**). Data shown are mean ± SEM. ***p < 0.001, ****p < 0.0001

We then studied the DNA methylation profile in offspring myocardium, which is one of the major mechanisms underlying programming effects. Among the various DNA methyltransferases (DNMTs), the DNMT1 expression significantly decreases (Fig. 3a), whereas DNMT3b expression increases in the myocardium of prenatally DEX-exposed male offspring (Fig. 3b), whereas they showed no changes in female offspring between DEX group and NS group (Figs. 3a, b). The other types of DNMTs, that is, DNMT3a and DNMT3L exhibited no significant changes, either in male or female offspring (Figs. 3c, d). As the DNMT1 and DNMT3b exhibited the opposite changes in myocardium of male offspring exposed to DEX during pregnancy, we then analyzed the global genomic DNA methylation status in offspring’s myocardium, and found that the global DNA methylation level was significantly increased in myocardium of prenatally DEX-exposed male offspring, but not in female offspring (Fig. 3e).

### MeDIP-seq results

**Fig. 4:**
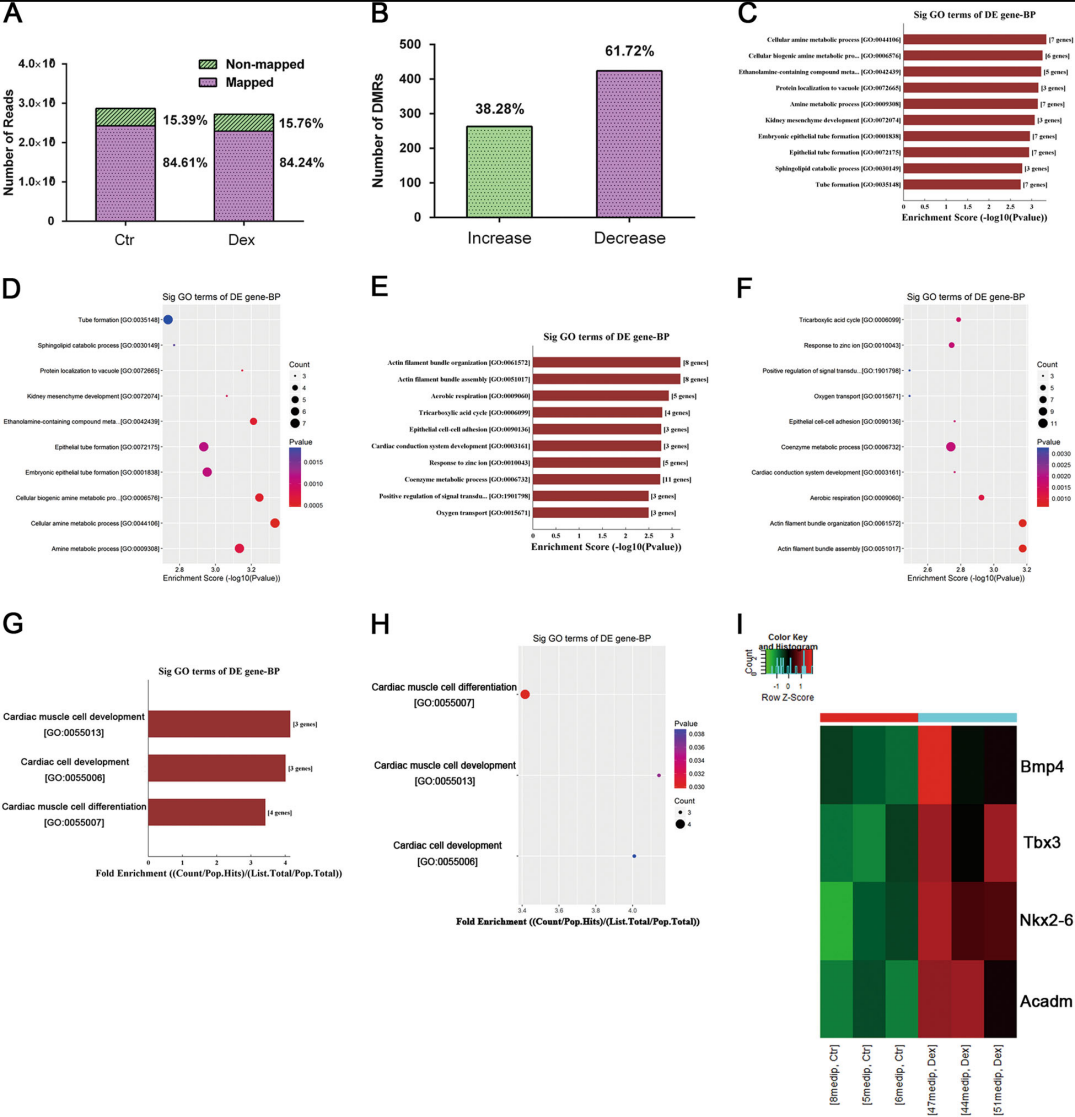
Differentially methylated loci in the DNA methylation profile and the pathway involved in cardiac muscle cell differentiation and development. **a** The total mapped peaks and non-mapped peaks in NS and DEX group. **b** The total number of significantly (log2-fold change ≥ 2) increased and decreased methylated genes in prenatally DEX-exposed offspring compared with prenatally NS-exposed offspring. **c**, **d** The top 10 GO biological processes with increased methylation. (**e**, **f**) The top 10 GO biological processes with decreased methylation. **g**, **h** The fold enrichment of the pathways involved in cardiac muscle cell differentiation and development with increased methylation. **i** The heatmap presented the methylation levels of individual genes involved in cardiac muscle cell differentiation and development. Red: upregulation; green: downregulation

To further identify changes in specific gene methylation patterns in the myocardium, whole-genome DNA methylation analysis was performed using the described MeDIP-seq method. The global differences in the DNA methylation profile between DEX-exposed rats and control rats are described in Fig. 4. We identified 24,280,328 mapped peaks and 4,416,301 non-mapped peaks from a total of 28,696,629 peaks in control rats with mapping ratio of 84.61%, and 22,921,630 mapped peaks and 4,287,699 non-mapped peaks from a total of 27,209,329 peaks in DEX-exposed rat with mapping ratio of 84.24% (Fig. 4a). A total of 687 DMRs had a ≥ 2-fold change (log2) in methylation in DEX-exposed rats compared with control rats, among which 263 DMRs (38.28%) exhibited increased methylation, and 424 (61.72%) DMRs exhibited decreased methylation (Fig. 4b). The top 50 genes with increased and decreased methylation levels based on log2-fold change are listed in supplemental Tables 1 and 2.

### GO functional and pathway analysis

To identify the biological function, networks and canonical pathways that was affected by the differentially methylated genes, we performed GO analysis after the MeDIP-seq analysis. In the analysis of genes with altered methylation ( ≥ 2-fold in log2) in DEX-exposed rat compared with control rat, the molecules with increased methylation and decreased methylation changes were categorized into 225 and 160 biological processes, respectively. The top 10 GO biological processes with increased methylation and decreased methylation were shown in Figs. 4c, d and Figs. 4e, f, respectively. Notably, molecules involved in the cardiac muscle cell differentiation and development pathway displayed both increase and decrease in methylation in the myocardium from DEX-exposed rats. Supplemental Tables 3 and 4 list the genes involved in the cardiac muscle cell differentiation and development pathway that exhibited altered methylation (4 genes with increased methylation in supplemental Table 3; 23 genes with decreased methylation in supplemental Table 4). The fold enrichment of the pathways involved in cardiac muscle cell differentiation and development with increased methylation is shown in Figs. 4g, h. More specifically, the methylation levels of the four genes (Bmp4, Tbx3, Acadm and Nkx2-6) are presented as the heatmap (Fig. 4i).

### BMP4 expression is significantly decreased in myocardium from male mice prenatally exposed to DEX

**Fig. 5:**
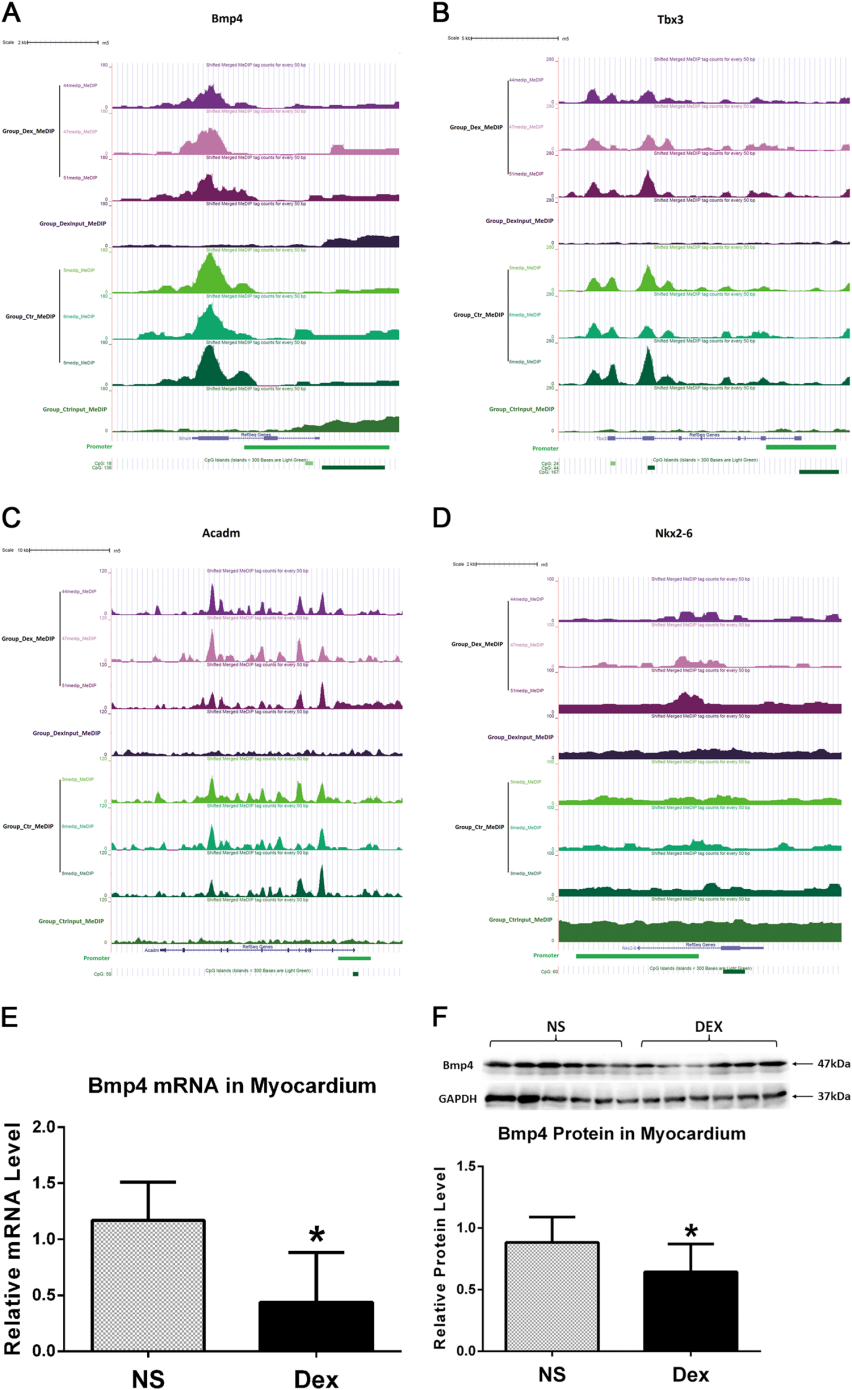
BMP4 expression is significantly decreased in myocardium from male offspring prenatally exposed to DEX. The methylated levels of the promoter and CpG island of the four genes involved in cardiac muscle cell differentiation and development, Bmp4 (**a**), Tbx3 (**b**), Acadm (**c**) and Nkx2-6 (**d**), were aligned and analyzed using publicly available software SignalMap. For each gene in each graph, the normalized number of reads covering each nucleotide position is shown in different tracks for Dex-treated (Group_Dex_MeDIP) or NS-treated (Group_Ctr_MeDIP) samples with MeDIP performed, each with three biological replicates, or for input samples (Group_DexInput_MeDIP and Group_CtrInput_MeDIP). The RefSeq gene models are shown in blue below the tracks, with arrowed lines showing introns and the direction of the gene on genome, narrow boxes for UTRs, and wide boxes indicating exons. The tracks below the gene model track are for the annotations of the promoter region, the CpG islands identified in the UCSC genome browser database. CpG islands in dark green are more reliable than those in light green as indicated. The Bmp4 mRNA (**e**) and protein (**f**) levels both were downregulated in myocardium from male mice prenatally exposed to DEX. Data shown are mean ± SEM. *p < 0.05

Sequences of the four genes with elevated methylation levels are processed and analyzed using publicly available software SignalMap (http://sequencing.roche.com), along with the corresponding gene’s promoter and CpG island (Figs. 5a-d). For each gene in each graph, the normalized number of reads covering each nucleotide position is shown in different tracks for Dex-treated (Group_Dex_MeDIP) or NS-treated (Group_Ctr_MeDIP) samples, or for input samples (Group_DexInput_MeDIP and Group_CtrInput_MeDIP). The RefSeq gene models are shown in blue below the tracks, with arrowed lines showing introns and the direction of the gene on genome, narrow boxes for UTRs (Untranslated Regions), and wide boxes indicating exons. The tracks below the gene model track are for the annotations of the promoter region, and the CpG islands identified in the UCSC genome browser database. CpG islands in dark green are more reliable than those in light green as indicated. It is shown that *Bmp4*, *Tbx3* and *Acadm* genes all contain CpG islands in their promoter regions, within which manifested the increased methylation levels in Group_Dex_MeDIP compared with Group_Ctr_MeDIP.

To further confirm the expression levels of these genes, quantitative reverse transcriptase-PCR and western blot were performed and we found that the mRNA and protein levels of bone morphogenetic protein-4 (Bmp4) were significantly decreased in myocardium from male mice prenatally exposed to DEX (Figs. 5e, f). Although methylation levels also change for the other three genes, their expression levels were not affected by prenatal DEX exposure (Supplemental Fig. 2A, B), suggesting BMP4 may play important roles in the programming effects of DEX on cardiac functions.

To further dissect the critical DNA elements in *Bmp4* locus responsible for methylation-dependent transcriptional regulation of *Bmp4* gene, we analyzed the promoter sequence of *Bmp4* for known transcription factor binding sites in the JASPAR2018 database (http://jaspar.genereg.net/) using the program MAST (version 4.11.2) in the MEME suite (http://meme-suite.org/). We identified 15 *cis*-elements for 12 different transcription factors across the promoter region of *Bmp4* (Supplemental Fig. 3). However, we failed to identify *cis*-elements that statistically significantly matched the known binding sites of GR/NR3C1.

### BMP4 inhibited H/R induced cardiomyocytes apoptosis in the male offspring prenatally exposed to DEX

**Fig. 6:**
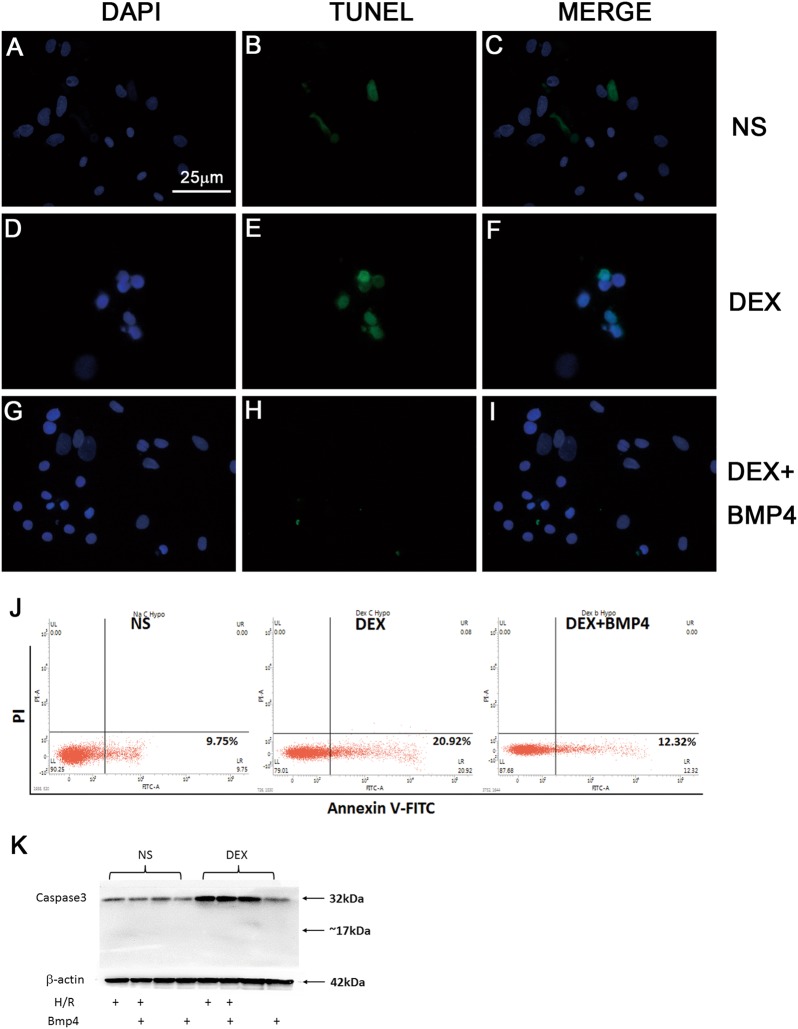
BMP4 rescued H/R induced cardiomyocytes apoptosis in the male offspring prenatally exposed to DEX. **a**-**c** TUNEL staining of the primary cardiomyocytes from the male offspring prenatally exposed to NS. **d**-**f** TUNEL staining of the primary cardiomyocytes from the male offspring prenatally exposed to DEX. **g**-**i** BMP4 pre-treatment (50 ng/ml) significantly decreased the number of apoptotic cardiomyocytes from prenatally DEX-exposed offspring induced by H/R. **j** Annexin V-PI double staining showed the ratio of early apoptosis of cardiomyocytes from each group with or without BMP4 pre-treatment. **k** The absence of cleaved caspase 3 in cardiomyocytes is consistent with Annexin V-PI double staining result that the late apoptosis or cell death was rare under such experimental settings

To further investigate the effects of BMP4 in mediating the programming effects of DEX on cardiomyocytes, we isolated and cultured the primary ventricular cells from 1-day-old rat pups, which were then undergone hypoxia/reoxygenation (H/R). In accordance with the results, we observed in myocardial tissues, the number of apoptotic cardiomyocytes was significantly increased in H/R rats prenatally exposed to DEX compared with that from prenatally NS-exposed control (Figs. 6a-f). Notably, BMP4 pre-treatment (50 ng/ml) significantly decreased the number of apoptotic cardiomyocytes induced by H/R in the prenatally DEX-exposed rats, indicated by TUNNEL staining (Fig. 6g-i). To further differentiate early apoptosis and late apoptosis, we analyzed the cardiomyocytes using flow cytometry with Annexin V-PI double staining, and found that only early apoptosis induced by H/R were significantly increased in the cardiomyocytes (20.92 vs. 9.75% in the 4th quadrant), whereas pre-treatment of BMP4 conferred resistance to H/R-induced apoptosis (12.32 vs. 20.92% in the 4th quadrant). In our experimental settings, the late apoptosis or cell death was rare (Fig. 6j). This was further confirmed by the absence of cleaved caspase 3 in cardiomyocytes, which is usually considered as an indicator of late apoptosis or cell death (Fig. 6k).

### DEX-enhanced apoptosis was dependent on mitochondria-derived ROS production and ameliorated by BMP4

**Fig. 7:**
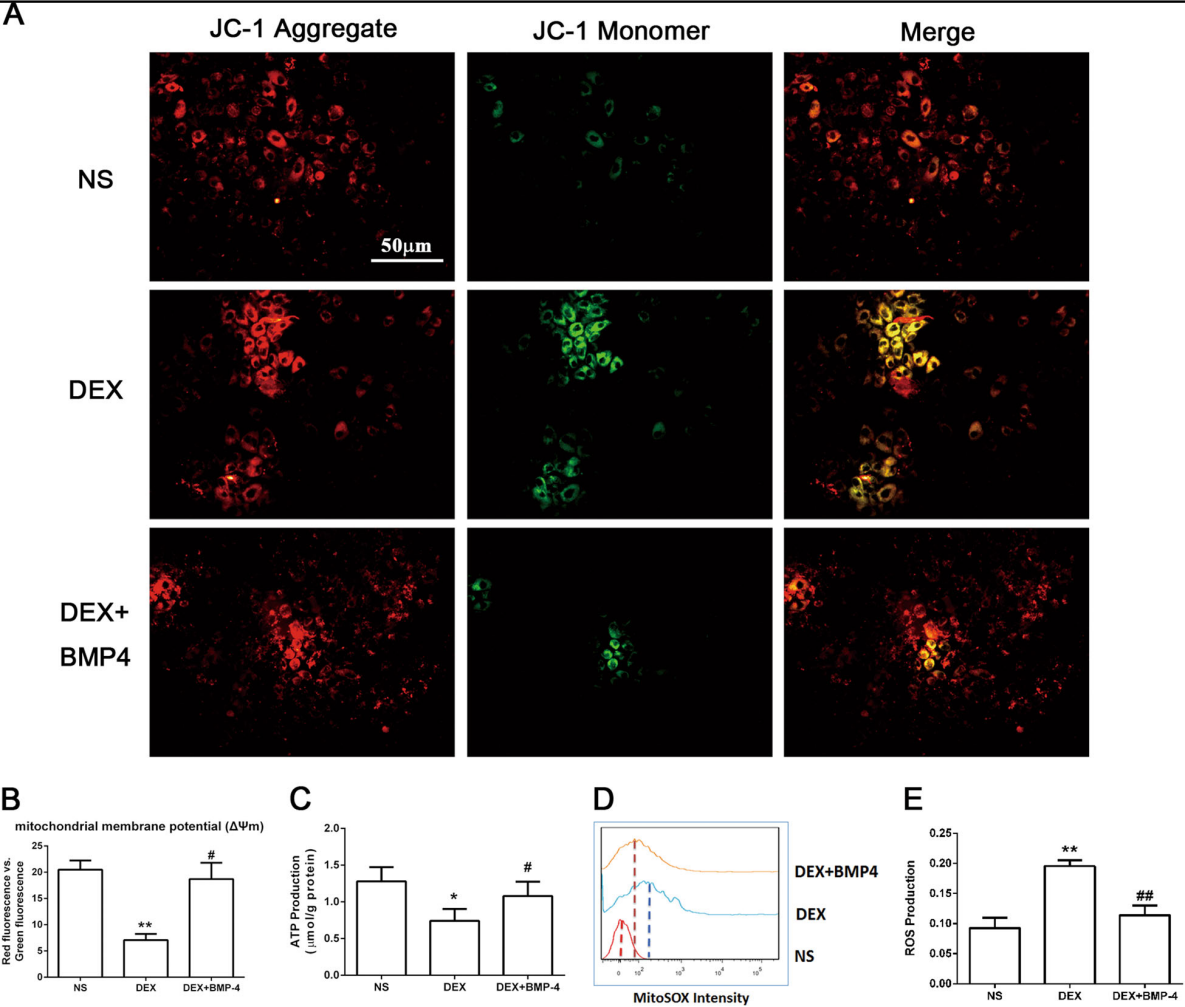
DEX-enhanced apoptosis is dependent on mitochondria-derived ROS production and could be rescued by BMP4 pre-treatment. Mitochondrial membrane potential (ΔΨm) was detected by JC-1 staining. When the mitochondrial membrane is intact, the JC-1 dye accumulates in mitochondria matrix and emits a red signal. When the mitochondrial membranes are damaged, the JC-1 dye diffuses into the cytoplasm and emits a green signal. **a** The myocardiocytes manifested significant increase in green fluorescence intensity after H/R from prenatally DEX-exposed offspring, compared with the prenatally NS-exposed control. This depletion of ΔΨm can be inhibited by the pre-treatment of BMP4 24 h prior to H/R. **b** The ratio of red absorbency vs green was quantified by a multifunction microplate reader. **c** ATP level, another indicator for mitochondrial functions, was significantly lower in the cardiomyocytes undergone H/R from rats prenatally exposed to DEX than those exposed to NS, and was restored by BMP4 pre-treatment. **d**, **e** Mito-SOX staining showed that the production of ROS was elevated in cardiomyocytes undergone H/R from rats prenatally exposed to DEX compared with those exposed to NS, and was significantly reversed in the presence of BMP4 prior to H/R. Data shown are mean ± SEM. *p < 0.05, **p < 0.01; ^#^*p* < 0.05, ^##^*p* < 0.01 compared with prenatally DEX-exposed male offspring

Cardiac cell apoptosis is closely associated with mitochondria dysfunction, which can be indicated by the breakdown of mitochondrial membrane potential (ΔΨm). To determine whether H/R-induced apoptosis in cardiomyocytes deteriorated by DEX involves mitochondrial damage, we measured the fluorescence emission shift (red to green) by a JC-1 dye kit. We found that DEX predisposing caused significant increase in green fluorescence intensity compared with the NS-exposed control, which can be inhibited by the pre-treatment of BMP4 24 h prior to H/R (Fig. 7a). The ratio of JC-1 aggregates positive cells (red) and monomer-positive cells (green) was quantified by multifunction microplate reader, demonstrating a remarkable reverse of ΔΨm by BMP4 as well (Fig. 7b). ATP level is another indicator for mitochondrial functions, which was significantly lower in the cardiomyocytes from DEX group than those from NS group. Again, BMP4 pre-treatment restored the production of ATP in cardiomyocytes undergone H/R (Fig. 7c).

The accumulation of ROS plays an important role in apoptosis induction and mitochondrial disruption under both physiologic and pathologic conditions^22^. So we next investigated the intracellular ROS generation in cardiomyocytes by Mito-SOX staining, which is a highly selective detector of superoxide in live cell mitochondria. Inversely consistent with ATP production, the intensity of Mito-SOX fluorescence was elevated in cardiomyocytes from DEX group compared with NS group by approximately 200-fold and was significantly decreased in the presence of BMP4 prior to H/R (Fig. 7d). Quantification of the fluorescence intensity confirmed that ROS generation was increased by DEX and mostly reversed by BMP4 pre-treatment, as well (Fig. 7e).

## Discussion

As the other extra-uterine adverse factors, such as hypoxia and malnutrition, could be reversed by the improvement of living conditions or diet supplement, the prenatal exposure to GCs is unavoidable under many circumstances. In the present study, we found that the ± dp/dt max was significantly decreased after I/R in the male offspring prenatally exposed to GC. This result indicated the myocardium of male offspring is more vulnerable to the “second hit”, that is, hypoxia/reperfusion injury, suggesting the prenatal GC exposure is one of the critical factors for the cardiac disorders in adulthood. The principle of later-life emergence is a major characteristic of developmental programming^23^. In accordance with this concept, we found that the ± dp/dt max were significantly decreased in male offspring undergone I/R at both 12 weeks and 24 weeks, corresponding to the early adult and relatively elder age. Although, cautions should be made to draw a conclusion that the programming effect on cardiac function lasts for the whole life of offspring. Also, to examine if these programming effects could be transmitted across generation or so called “transgenerationally”, the F3 and beyond offspring need to be studied in the future work. Due to the ease of genetic manipulation in small animal models^24^, we are planning to utilize the tissue-specific transgenic mice in our future work to reveal the mechanisms underpinning the discovered target genes. However, the rodent models also present limitations, in particular the extent to which it is possible to translate specific findings into human pregnancy as rodents are altricial species, born at an earlier stage of development than the human infant^25^. Therefore, careful consideration needs to be taken to optimize effective translation to the human setting.

The sex-specific effects of prenatal GC exposure have been reported in many organs before^26–29^. Previous studies have shown that female offspring are resistant to prenatal hypoxia-induced and maternal high-fat diet (HFD)-induced programming in myocardial vulnerability to I/R injury^30,31^. Observational studies discovered that premenopausal women tend to be more resistant to cardiovascular disease compare with age-matched men, with the risk increases after the onset of menopause^32,33^. Mechanically, prenatal hypoxia and maternal HFD resulted in significantly increased angiotensin II type 2 receptor (Agtr2) mRNA and protein abundance in male, but not in female offspring^31,34^. OVX decreased the expression of Agtr1 and increased Agtr2 expression in the heart, which were abrogated by E_2_ replacement^35^, suggesting that estrogen plays a role in protecting females in fetal programming from increased heart vulnerability. There is evidence that male rat offspring are more susceptible to developmentally programmed hypertension than female offspring intervened with antenatal DEX administration^36^. In human studies, placental 11β-hydroxysteroid dehydrogenase-2 activity in response to antenatal steroid administration has been observed to increase specifically in female offspring^37^, which would protect the female offspring from GC insults and confer advantage in neonatal outcome. However, the direct effect of DEX is to induce the protection in rat hearts from I/R-mediated injury^30^, which is contradictory to our findings. These controversial results suggested the different mechanisms underlying direct effects and programming effects mediated through maternal-fetal crosstalk of GCs.

It was shown that prenatal synthetic GC treatment changes DNA methylation states only in male organ systems^38^, consistent with our previous findings that the expression of serum- and GC-regulated protein kinase 1 (Sgk1) were decreased in male offspring’s hearts exposed to excess GCs prenatally, with the proximal CpG island in the Sgk1 promoter hypermethylated. These studies suggested that the sex-specific programming effects of GC may be mediated by the epigenetic regulation of DNA methylation in offspring myocardium. The expression pattern of Dnmts have been shown highly dynamic and organ specific in fetal kidneys and placentas during gestation, as well as in adult offspring kidneys and cerebellum^38^. Intriguingly, Dnmt1 and Dnmt3b manifested opposite expression in the myocardium of male offspring prenatally exposed to GC, whereas Dnmt3a and Dnmt3l showed no changes. However, to further reveal estrogen/ER signaling pathways interfering the expression of DNMTs so that contributing to the steady of methylation state in female myocardium, is of great interest for the future investigation. The effects of GC on specific gene promoters were reported in many studies^39–41^, but the gene modification in cardiac tissues was rarely investigated. Our study firstly conducted genome-wide MeDIP sequencing on offspring’s myocardium and revealed the epigenetic modification of promoters in specific genes involved in cardiac muscle cell differentiation and development pathway.

Among the four genes whose promoters were hypermethylated in myocardium, only Bmp4 expression was confirmed to be decreased, which is inversely correlated with its promoter’s methylation level. The classical view of methylation-mediated protein–DNA interactions is that only proteins with a methyl-CpG binding domain (MBD), including MeCP2 (methyl-CpG-binding protein 2), MBD1, MBD2 and MBD4, can interact with methylated DNA in a non-sequence-specific manner^42,43^. However, evidence is emerging to suggest that transcription factors lacking a MBD can also interact with methylated DNA, in a sequence-dependent manner^44^. As neither known nor putative binding sites of GR/NR3C1 were identified across the promoter region of *Bmp4* responsible for methylation-dependent transcriptional regulation, it would be of great interest to investigate in the future if the regulation of *Bmp4* by GR could be indirect, possibly via cooperation with the TFs shown in Supplemental Fig. 3, such as FOXA2 and NFYB, by binding to the specific methylated sequences in *Bmp4* promoter.

BMPs are multifunctional growth factors belonging to the transforming growth factor-beta superfamily and comprise a subfamily of >20 members^45^, involved in the regulation of cell proliferation, survival, differentiation and apoptosis^46^. Recently, BMP4 has been found to play a pivotal role in the development of fetal heart^45,47^. Meanwhile, BMP4 was broadly used to induce the cardiomyocytes from human pluripotent stem cells^48,49^. However, the functions of BMP4 in adult heart was ambiguous. BMP4 induced the hypertrophy, apoptosis and fibrosis of cardiomyocytes in vitro, which can be reversed by BMP4 inhibitors Noggin and DMH1^50^. Pachori et al. reported that BMP4 mediates myocardial ischemic injury through JNK(c-Jun N-terminal kinase)-dependent signaling pathway^51^. On the contrary, it was also found that human recombinant BMP4 promoted survival after H_2_O_2_ injury in HL-1 cells, and also protected adult mouse cardiomyocytes against hypoxia-reoxygenation injury^47^. Moreover, BMP4 exerts prooxidant, prohypertensive and proinflammatory effects in the systemic circulation, whereas pulmonary arteries are protected from these adverse effects of BMP4^52^. In the present study, we clearly showed that pre-treatment of BMP4 conferred resistance to H/R-induced apoptosis in the prenatally DEX-exposed rats, suggesting the timing, duration, local concentration, and maybe more importantly, the downstream signaling of BMP4 are of importance to the fetal heart development and the functions of adult heart^45,47^. In the future work, by specifically inducing the BMP4 expression in the myocardium using cardiac-specific BMP4 knock-in mouse model, the roles of BMP4 in the cardiac functions, as well as its involvement in the effects of prenatal GC exposure on offspring’s hearts will be further defined.

BMPs bind with two types of receptors to form heteromeric complex, which subsequently phosphorylate SMAD1, SMAD5 or SMAD8 that is recruited into the receptor complex to convey the signal intracellularly^45^. Besides the classic pathways mediated by SMADs (drosophila mothers against decapentaplegic protein), BMP signaling can be also mediated by MAP3K7/MAP3K7IP1 leading to the activation of p38 MAPK (mitogen-activated protein kinase), PI3 kinase (phosphatidylinositol 3-kinase), RAS (small G-protein), ERK (extracellular regulated protein kinase), and JNK^53,54^, showing a broad diversities of BMPs downstream signaling pathways. A recent study showed that another member of BMP family, BMP-7, facilitates the recovery of cardiac function after acute myocardial infarction through attenuating TGF-β1 (transforming growth factor-β1) and its downstream signaling pathway Smad2/3^55^. Moreover, BMP signaling downregulates transcription of the miRNA-302~367 gene cluster and depresses the expression of the type II BMP receptor, which forms a negative regulatory loop to potentially maintain the BMP signaling pathway^56^. Upon these fine-tuning BMP signaling regulated both positively and negatively by extrinsic and intrinsic regulatory factors, future work is apparently warrant to dissect the specific downstream signaling pathway that mediates the protective effects of BMP4 on the I/R injury of cardiomyocytes in the offspring prenatally exposed to excess GC.

Accumulating evidence show that oxidative stress is present in cardiovascular diseases, such as atherosclerosis, vascular dysfunction and vascular remodeling^57–59^. Mitochondria can be an important source of superoxide and also themselves targets of ROS, leading to mitochondrial dysfunction and exaggerating ROS production^60^. The current study observed that the release of ROS and dysfunction of mitochondria might form a vicious cycle to substantially contribute to the apoptosis of cardiomyocytes in male offspring prenatally exposed to GC, upon I/R injury. Whether the decrease of BMP4 causes the reduced mitochondrial DNA content in myocardium or influences any mitochondria-specific protein expression need to be further investigated in the future study.

## Conclusions

**Fig. 8:**
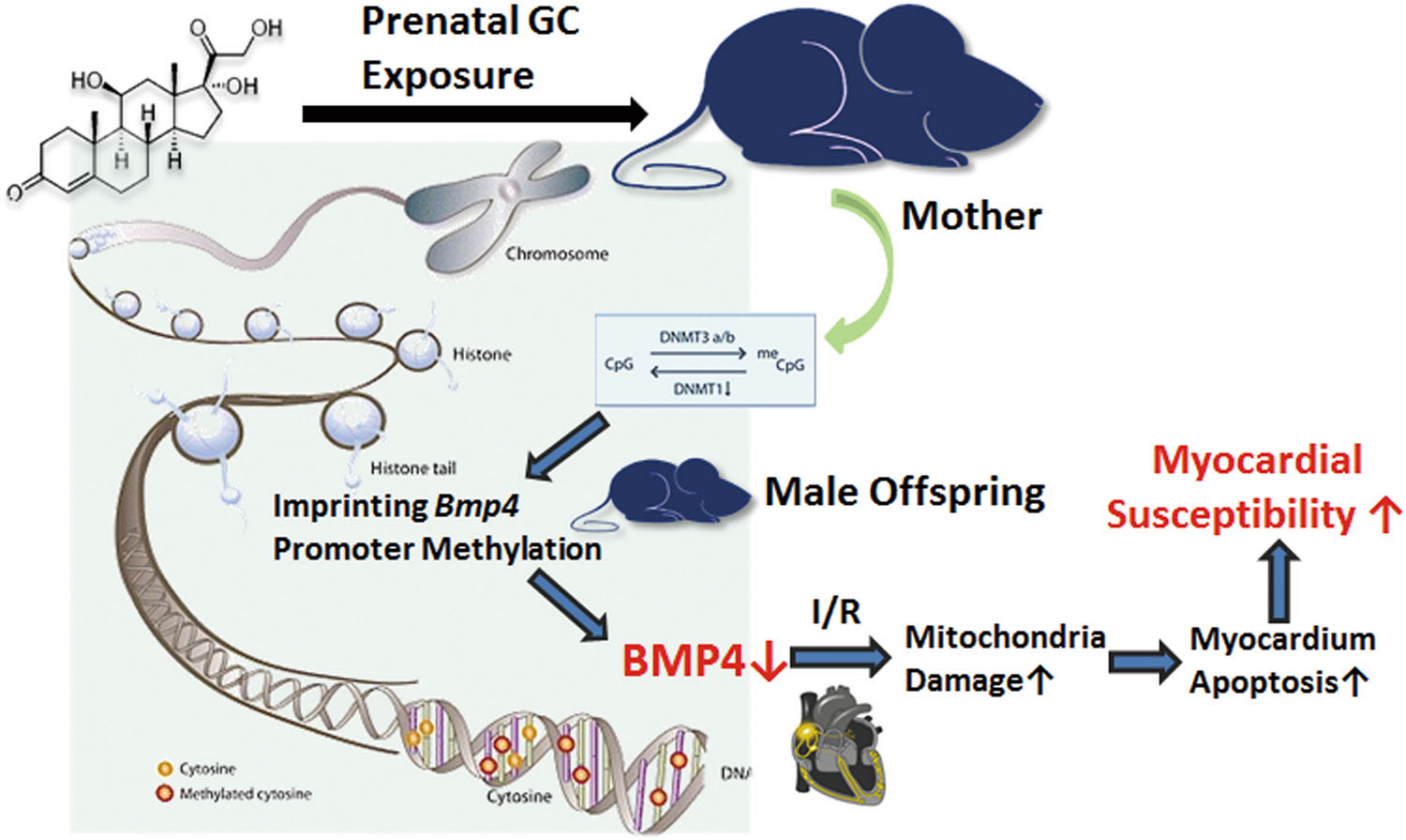
Working model of the GC programming effects on heart

Our findings suggested that the prenatal GC exposure increases the myocardium susceptibility of male offspring to postnatal injury due to the decrease of protective factor BMP4 (Fig. 8). Under the clinical settings that GC application is inevitable, the addition of exogenous BMP4 may provide a promising treatment for the congenital heart diseases.

## Electronic supplementary material

Supplemental Data

## References

[1] Gluckman PD, Hanson MA, Cooper C, Thornburg KL (2008). Effect of in utero and early-life conditions on adult health and disease. N. Engl. J. Med..

[2] Barker DJ, Osmond C (1986). Infant mortality, childhood nutrition, and ischaemic heart disease in England and Wales. Lancet.

[3] Harris A, Seckl J (2011). Glucocorticoids, prenatal stress and the programming of disease. Horm. Behav..

[4] Santos MS, Joles JA (2012). Early determinants of cardiovascular disease. Best Pract. Res. Clin. Endocrinol. Metab..

[5] Moisiadis VG, Matthews SG (2014). Glucocorticoids and fetal programming part 1: outcomes. Nat. Rev. Endocrinol..

[6] Patterson AJ, Chen M, Xue Q, Xiao D, Zhang L (2010). Chronic prenatal hypoxia induces epigenetic programming of PKC{epsilon} gene repression in rat hearts. Circ. Res..

[7] Kawamura M (2007). Undernutrition in utero augments systolic blood pressure and cardiac remodeling in adult mouse offspring: possible involvement of local cardiac angiotensin system in developmental origins of cardiovascular disease. Endocrinology.

[8] Fowden AL, Li J, Forhead AJ (1998). Glucocorticoids and the preparation for life after birth: are there long-term consequences of the life insurance?. Proc. Nutr. Soc..

[9] Gao L (2015). Steroid receptor coactivators 1 and 2 mediate fetal-to-maternal signaling that initiates parturition. J. Clin. Invest..

[10] Van Vliet G, Polak M, Ritzen EM (2008). Treating fetal thyroid and adrenal disorders through the mother. Nat. Clin. Pract. Endocrinol. Metab..

[11] Namazy JA, Schatz M (2005). Treatment of asthma during pregnancy and perinatal outcomes. Curr. Opin. Allergy Clin. Immunol..

[12] Doria A, Tincani A, Lockshin M (2008). Challenges of lupus pregnancies. Rheumatology.

[13] Grgic O, Ivanisevic M, Delmis J (2010). Treatment of idiopathic thrombocytopenic purpura in pregnancy with pulsed dose of dexamethasone. J. Obstet. Gynaecol..

[14] Wyrwoll CS, Mark PJ, Waddell BJ (2007). Developmental programming of renal glucocorticoid sensitivity and the renin-angiotensin system. Hypertension.

[15] Bogdarina I, Haase A, Langley-Evans S, Clark AJ (2010). Glucocorticoid effects on the programming of AT1b angiotensin receptor gene methylation and expression in the rat. PLoS One.

[16] Sheen JM (2015). Prenatal dexamethasone-induced programmed hypertension and renal programming. Life. Sci..

[17] Seckl JR, Meaney MJ (2004). Glucocorticoid programming. Ann. N. Y. Acad. Sci..

[18] Schiller NB (1989). Recommendations for quantitation of the left ventricle by two-dimensional echocardiography. American Society of Echocardiography Committee on Standards, Subcommittee on Quantitation of Two-Dimensional Echocardiograms. J. Am. Soc. Echocardiogr..

[19] Yao LL (2010). Hydrogen sulfide protects cardiomyocytes from hypoxia/reoxygenation-induced apoptosis by preventing GSK-3beta-dependent opening of mPTP. Am. J. Physiol. Heart Circ. Physiol..

[20] Taylor PD (2018). Generation of maternal obesity models in studies of developmental programming in rodents. Methods Mol. Biol..

[21] Livak KJ, Schmittgen TD (2001). Analysis of relative gene expression data using real-time quantitative PCR and the 2(-Delta Delta C(T)) method. Methods.

[22] Zhao P (2015). Angiotensin1-7 protects cardiomyocytes from hypoxia/reoxygenation-induced oxidative stress by preventing ROS-associated mitochondrial dysfunction and activating the Akt signaling pathway. Acta Histochem..

[23] Reynolds CM, Vickers MH (2018). Utility of small animal models of developmental programming. Methods Mol. Biol..

[24] Rabadan-Diehl C, Nathanielsz P (2013). From mice to men: research models of developmental programming. J. Dev. Orig. Health Dis..

[25] Poston L (2010). Developmental programming and diabetes - the human experience and insight from animal models. Best Pract. Res. Clin. Endocrinol. Metab..

[26] Su Y (2015). Antenatal glucocorticoid treatment alters Na+ uptake in renal proximal tubule cells from adult offspring in a sex-specific manner. Am. J. Physiol. Ren. Physiol..

[27] O’Sullivan L (2015). Excess prenatal corticosterone exposure results in albuminuria, sex-specific hypotension, and altered heart rate responses to restraint stress in aged adult mice. Am. J. Physiol. Ren. Physiol..

[28] Bi J (2014). Sex-specific effect of antenatal betamethasone exposure on renal oxidative stress induced by angiotensins in adult sheep. Am. J. Physiol. Ren. Physiol..

[29] Aiken CE, Ozanne SE (2013). Sex differences in developmental programming models. Reproduction.

[30] Xue Q, Zhang L (2009). Prenatal hypoxia causes a sex-dependent increase in heart susceptibility to ischemia and reperfusion injury in adult male offspring: role of protein kinase C epsilon. J. Pharmacol. Exp. Ther..

[31] Xue Q., et al. Maternal high-fat diet causes a sex-dependent increase in AGTR2 expression and cardiac dysfunction in adult male rat offspring. *Biol. Reprod.* **93**, 2 (2015). 10.1095/biolreprod.115.129916 26157067

[32] Mendelsohn ME (2002). Protective effects of estrogen on the cardiovascular system. Am. J. Cardiol..

[33] Mendelsohn ME, Karas RH (1999). The protective effects of estrogen on the cardiovascular system. N. Engl. J. Med..

[34] Xue Q, Dasgupta C, Chen M, Zhang L (2011). Foetal hypoxia increases cardiac AT(2)R expression and subsequent vulnerability to adult ischaemic injury. Cardiovasc. Res..

[35] Xue Q, Xiao D, Zhang L (2015). Estrogen regulates angiotensin II receptor expression patterns and protects the heart from ischemic injury in female rats. Biol. Reprod..

[36] Ortiz LA, Quan A, Zarzar F, Weinberg A, Baum M (2003). Prenatal dexamethasone programs hypertension and renal injury in the rat. Hypertension.

[37] Stark MJ, Wright IM, Clifton VL (2009). Sex-specific alterations in placental 11beta-hydroxysteroid dehydrogenase 2 activity and early postnatal clinical course following antenatal betamethasone. Am. J. Physiol. Regul. Integr. Comp. Physiol..

[38] Crudo A (2012). Prenatal synthetic glucocorticoid treatment changes DNA methylation states in male organ systems: multigenerational effects. Endocrinology.

[39] Petropoulos S, Matthews SG, Szyf M (2014). Adult glucocorticoid exposure leads to transcriptional and DNA methylation changes in nuclear steroid receptors in the hippocampus and kidney of mouse male offspring. Biol. Reprod..

[40] Bockmuhl Y (2015). Methylation at the CpG island shore region upregulates Nr3c1 promoter activity after early-life stress. Epigenetics.

[41] Conradt E, Lester BM, Appleton AA, Armstrong DA, Marsit CJ (2013). The roles of DNA methylation of NR3C1 and 11beta-HSD2 and exposure to maternal mood disorder in utero on newborn neurobehavior. Epigenetics..

[42] Hendrich B, Tweedie S (2003). The methyl-CpG binding domain and the evolving role of DNA methylation in animals. Trends Genet..

[43] Jaenisch R, Bird A (2003). Epigenetic regulation of gene expression: how the genome integrates intrinsic and environmental signals. Nat. Genet..

[44] Zhu H, Wang G, Qian J (2016). Transcription factors as readers and effectors of DNA methylation. Nat. Rev. Genet..

[45] van Wijk B, Moorman AF, van den Hoff MJ (2007). Role of bone morphogenetic proteins in cardiac differentiation. Cardiovasc. Res..

[46] Xiao YT, Xiang LX, Shao JZ (2007). Bone morphogenetic protein. Biochem. Biophys. Res. Commun..

[47] Wu X (2014). Expression of bone morphogenetic protein-4 and its receptors in the remodeling heart. Life Sci..

[48] Palpant NJ (2017). Generating high-purity cardiac and endothelial derivatives from patterned mesoderm using human pluripotent stem cells. Nat. Protoc..

[49] van den Berg CW, Elliott DA, Braam SR, Mummery CL, Davis RP (2016). Differentiation of human pluripotent stem cells to cardiomyocytes under defined conditions. Methods Mol. Biol..

[50] Sun B (2013). Bone morphogenetic protein-4 mediates cardiac hypertrophy, apoptosis, and fibrosis in experimentally pathological cardiac hypertrophy. Hypertension.

[51] Pachori AS (2010). Bone morphogenetic protein 4 mediates myocardial ischemic injury through JNK-dependent signaling pathway. J. Mol. Cell. Cardiol..

[52] Csiszar A, Labinskyy N, Jo H, Ballabh P, Ungvari Z (2008). Differential proinflammatory and prooxidant effects of bone morphogenetic protein-4 in coronary and pulmonary arterial endothelial cells. Am. J. Physiol. Heart Circ. Physiol..

[53] Nohe A, Keating E, Knaus P, Petersen NO (2004). Signal transduction of bone morphogenetic protein receptors. Cell. Signal..

[54] Roux PP, Blenis J (2004). ERK and p38 MAPK-activated protein kinases: a family of protein kinases with diverse biological functions. Microbiol. Mol. Biol. Rev..

[55] Jin Y, Cheng X, Lu J, Li X (2018). Exogenous BMP-7 facilitates the recovery of cardiac function after acute myocardial infarction through counteracting TGF-beta1 signaling pathway. Tohoku J. Exp. Med..

[56] Kang H (2012). Inhibition of microRNA-302 (miR-302) by bone morphogenetic protein 4 (BMP4) facilitates the BMP signaling pathway. J. Biol. Chem..

[57] Li JM, Shah AM (2004). Endothelial cell superoxide generation: regulation and relevance for cardiovascular pathophysiology. Am. J. Physiol. Regul. Integr. Comp. Physiol..

[58] Paravicini TM, Touyz RM (2006). Redox signaling in hypertension. Cardiovasc. Res..

[59] Majzunova M, Dovinova I, Barancik M, Chan JY (2013). Redox signaling in pathophysiology of hypertension. J. Biomed. Sci..

[60] Ballinger SW (2000). Hydrogen peroxide- and peroxynitrite-induced mitochondrial DNA damage and dysfunction in vascular endothelial and smooth muscle cells. Circ. Res..

