# Supplementary material for: The detrimental effects of glucocorticoids exposure during pregnancy on offspring’s cardiac functions mediated by hypermethylation of bone morphogenetic protein-4
Source: Cell Death Dis. 2018 Aug 6;9(8):834. doi: 10.1038/s41419-018-0841-1 (PMC6079031; doi:10.1038/s41419-018-0841-1)
Supplement: Supplementary file 1 — Supplemental Data [file 41419_2018_841_MOESM1_ESM.docx]

**

**

**Supplemental Fig. 1 Cardiac functions were compromised in prenatally DEX-exposed adult male offspring at 24 weeks old.** The LVEF (A) and LVFS (B) did not manifest significant changes in the male or female offspring exposed to DEX during late gestation compared with that exposed to NS. The LV dp/dt max (C) and LV -dp/dt max (D) both significantly decreased after ischemia-reperfusion (I/R) injury in adult male offspring but not in adult female offspring, which were prenatally exposed to DEX. NS, normal saline; DEX, dexamethasone; LVEF, left ventricular ejection fraction; LVFS, left ventricular fractional shortening; dp/dt max, maximal rate of left ventricle systolic pressure change; -dp/dt max, maximal rate of left ventricle diastolic pressure change. Data shown are mean ± SEM. *P< 0.05, **P<0.01.

**

**

**Supplemental Fig. 2 The expression of other genes involved in cardiac muscle cell differentiation and development.** Western blots for the indicated proteins Acadm (A) and Tbx3 (B) in myocardium from prenatally DEX-exposed male offspring compared with NS exposed group. Immunoblots were shown in the upper panel. Densitometry analyses for Acadm and Tbx3 were expressed relative to the loading control, GADPH, in the lower panel.

**
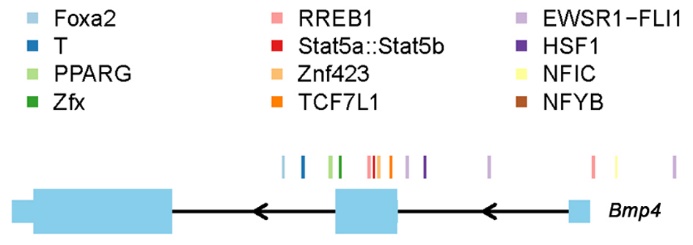
**

**Supplemental Fig. 3 Transcription factor binding sites (TFBSs) identified in the promoter region of BMP4.** Matrices for known vertebrate TFBSs in the JASPAR2018 database (http://jaspar.genereg.net/) were searched against the promoter sequence of BMP4 using the program MAST (version 4.11.2) in the MEME suite (http://meme-suite.org/). The top 15 hits with position p-value<1e-5 for 12 different TFs are shown as short vertical lines above the gene model for BMP4, which is shown below, with wide boxes in blue indicating exons, narrow blue boxes for UTRs, black lines for introns, and arrows showing the direction of the gene on genome, in the same as in Figure 5A-D.

**Supplemental Table 1. Top 50 annotated genes with increased methylation**

| **Rank** | **Symbol** | **Gene name** | **log_2_ Fold Change** | **Accession Number** |
| --- | --- | --- | --- | --- |
| 1 | Tmem128 | transmembrane protein 128 | 5.26 | NM_001108362 |
| 2 | Rab3c | ras-related protein Rab-3C | 5.01 | NM_133536 |
| 3 | Ngly1 | peptide-N(4)-(N-acetyl-beta- glucosaminyl)asparagine amidase | 4.95 | NM_001014136 |
| 4 | Mc5r | melanocortin receptor 5 | 4.95 | NM_013182 |
| 5 | Zfp39 | zinc finger protein 39 | 4.87 | NM_001107004 |
| 6 | RGD1310212 | uncharacterized protein LOC299557 | 4.87 | NM_001106765 |
| 7 | LOC690918 | uncharacterized protein LOC690918 | 4.87 | NM_001109613 |
| 8 | Pex11a | peroxisomal membrane protein 11A | 4.82 | NM_053487 |
| 9 | Ifi35 | interferon-induced protein 35 | 4.82 | NM_001009625 |
| 10 | Ddx39a | ATP-dependent RNA helicase DDX39A | 4.81 | NM_053563 |
| 11 | Olr1619 | olfactory receptor Olr1619 | 4.8 | NM_001000521 |
| 12 | Olr837 | olfactory receptor Olr837 | 4.75 | NM_001000897 |
| 13 | Gpsm2 | G-protein-signaling modulator 2 | 4.73 | NM_001191962 |
| 14 | Msmb | beta-microseminoprotein precursor | 4.71 | NM_019188 |
| 15 | Frmd3 | FERM domain-containing protein 3 | 4.68 | NM_001106662 |
| 16 | LOC680549 | homeobox protein PKNOX2 isoform 2 | 4.67 | NM_001271279 |
| 17 | Slc38a9 | putative sodium-coupled neutral amino acid transporter 9 | 4.66 | NM_001035251 |
| 18 | Sh3bp5 | SH3 domain-binding protein 5 | 4.66 | NM_054011 |
| 19 | Trim6 | tripartite motif-containing 6 | 4.65 | NM_001170461 |
| 20 | Cyp2c11 | cytochrome P450 2C11 precursor | 4.65 | NM_019184 |
| 21 | Abhd12 | monoacylglycerol lipase ABHD12 | 4.65 | NM_001024314 |
| 22 | Stam | signal transducing adapter molecule 1 | 4.64 | NM_001109121 |
| 23 | Lin7a | protein lin-7 homolog A | 4.64 | NM_053514 |
| 24 | Olr13 | olfactory receptor Olr13 | 4.61 | NM_001000116 |
| 25 | Gpn2 | GPN-loop GTPase 2 | 4.58 | NM_001270959 |
| 26 | Nudc | nuclear migration protein nudC | 4.54 | NM_017271 |
| 27 | Mapkapk3 | MAP kinase-activated protein kinase 3 | 4.54 | NM_001012127 |
| 28 | Slc2a9 | solute carrier family 2, facilitated glucose transporter member 9 | 4.5 | NM_001191551 |
| 29 | Ganc | neutral alpha-glucosidase C | 4.5 | NM_001145840 |
| 30 | Aldh8a1 | aldehyde dehydrogenase family 8 member A1 | 4.5 | NM_001191088 |
| 31 | Smarcc1 | SWI/SNF complex subunit SMARCC1 | 4.48 | NM_001106861 |
| 32 | Rnf165 | ring finger protein 165 | 4.48 | NM_001164505 |
| 33 | Phf1 | PHD finger protein 1 | 4.45 | NM_212538 |
| 34 | Efcc1 | EF-hand domain-containing protein ENSP00000381169 homolog | 4.44 | NM_001163921 |
| 35 | Fbln1 | fibulin-1 precursor | 4.38 | NM_001127547 |
| 36 | Tmem2 | transmembrane protein 2 | 4.37 | NM_001107596 |
| 37 | Ctgf | connective tissue growth factor precursor | 4.35 | NM_022266 |
| 38 | Enpp6 | ectonucleotide pyrophosphatase/phosphodiesterase family member 6 precursor | 4.33 | NM_001107311 |
| 39 | Hoxa6 | homeobox protein Hox-A6 | 4.29 | NM_001191087 |
| 40 | Olr341 | olfactory receptor Olr341 | 4.27 | NM_001001042 |
| 41 | Klhl15 | kelch-like protein 15 | 4.21 | NM_001108021 |
| 42 | Guca2a | guanylin precursor | 4.21 | NM_013118 |
| 43 | Dgkb | diacylglycerol kinase beta | 4.21 | NM_019304 |
| 44 | Ppm1g | protein phosphatase 1G | 4.2 | NM_147209 |
| 45 | Olr1332 | olfactory receptor Olr1332 | 4.2 | NM_001000477 |
| 46 | Csrnp3 | cysteine/serine-rich nuclear protein 3 | 4.2 | NM_001271225 |
| 47 | Olr1533 | olfactory receptor Olr1533 | 4.18 | NM_001000496 |
| 48 | Dph3 | zinc finger, CSL domain containing 2 | 4.14 | NM_001134850 |
| 49 | Cab39l | calcium-binding protein 39-like | 4.14 | NM_001011917 |
| 50 | Lamtor4 | late endosomal/lysosomal adaptor, MAPK and MTOR activator 4 | 4.13 | NM_001108330 |

**Supplemental Table 2 Top 50 annotated genes with decreased methylation**

| **Rank** | **Symbol** | **Gene name** | **log_2_ Fold Change** | **Accession Number** |
| --- | --- | --- | --- | --- |
| 1 | Slc44a1 | choline transporter-like protein 1 isoform 1 | -5 | NM_001033852 |
| 2 | Vps13d | vacuolar protein sorting-associated protein 13D | -4.94 | NM_001108006 |
| 3 | Bbs2 | Bardet-Biedl syndrome 2 protein homolog | -4.87 | NM_053618 |
| 4 | Ell | RNA polymerase II elongation factor ELL | -4.86 | NM_001107304 |
| 5 | Slamf6 | SLAM family member 6 | -4.78 | NM_001191932 |
| 6 | Mrgpre | mas-related G-protein coupled receptor member E | -4.77 | NM_001002288 |
| 7 | Zfp157 | zinc finger protein 157 | -4.65 | NM_001170404 |
| 8 | Kpna2 | importin subunit alpha-2 | -4.54 | NM_053483 |
| 9 | Seli | ethanolaminephosphotransferase 1 | -4.51 | NM_001134754 |
| 10 | Rhebl1 | GTPase RhebL1 precursor | -4.49 | NM_182825 |
| 11 | Cpt2 | carnitine O-palmitoyltransferase 2, mitochondrial precursor | -4.49 | NM_012930 |
| 12 | Cnot1 | CCR4-NOT transcription complex subunit 1 | -4.49 | NM_001134840 |
| 13 | Apitd1 | centromere protein S | -4.48 | NM_001204879 |
| 14 | Tmem55b | transmembrane protein 55B | -4.47 | NM_001014233 |
| 15 | LOC684871 | uncharacterized protein LOC684871 | -4.45 | NM_001115043 |
| 16 | Cdk11b | cyclin-dependent kinase 11B | -4.45 | NM_145766 |
| 17 | Lgi1 | leucine-rich glioma-inactivated protein 1 precursor | -4.44 | NM_145769 |
| 18 | Sik1 | serine/threonine-protein kinase SIK1 | -4.43 | NM_021693 |
| 19 | Chpf2 | chondroitin sulfate glucuronyltransferase precursor | -4.43 | NM_001106574 |
| 20 | RGD1561517 | uncharacterized protein LOC499927 | -4.42 | NM_001135901 |
| 21 | Rom1 | rod outer segment membrane protein 1 | -4.41 | NM_001009690 |
| 22 | RGD1565033 | uncharacterized protein LOC498014 | -4.41 | NM_001109050 |
| 23 | LOC688318 | uncharacterized protein LOC688318 | -4.36 | NM_001127571 |
| 24 | Ttc9c | tetratricopeptide repeat protein 9C | -4.35 | NM_001007693 |
| 25 | Fgf23 | fibroblast growth factor 23 precursor | -4.35 | NM_130754 |
| 26 | Adam15 | disintegrin and metalloproteinase domain-containing protein 15 precursor | -4.35 | NM_020308 |
| 27 | Trappc5 | trafficking protein particle complex subunit 5 | -4.33 | NM_001108850 |
| 28 | Sgca | alpha-sarcoglycan precursor | -4.33 | NM_001107039 |
| 29 | Haus1 | HAUS augmin-like complex subunit 1 | -4.33 | NM_138864 |
| 30 | Suv420h2 | histone-lysine N-methyltransferase SUV420H2 | -4.32 | NM_001107475 |
| 31 | St3gal4 | CMP-N-acetylneuraminate-beta-galactosamide- alpha-2,3-sialyltransferase 4 | -4.3 | NM_203337 |
| 32 | Ppfia1 | protein tyrosine phosphatase, receptor type, f polypeptide (PTPRF), interacting protein, alpha 1 | -4.29 | NM_001106320 |
| 33 | Gpt | alanine aminotransferase 1 | -4.25 | NM_031039 |
| 34 | Nr1d1 | nuclear receptor subfamily 1 group D member 1 isoform 1 | -4.24 | NM_001113422 |
| 35 | Spata5 | spermatogenesis-associated protein 5 | -4.22 | NM_001108549 |
| 36 | Fgd2 | FYVE, RhoGEF and PH domain-containing protein 2 | -4.2 | NM_001107617 |
| 37 | Copz2 | coatomer subunit zeta-2 | -4.18 | NM_001108294 |
| 38 | Olr1202 | olfactory receptor Olr1202 | -4.16 | NM_001000817 |
| 39 | Mrgprb13 | mas-related G-protein coupled receptor member B8 | -4.13 | NM_001002283 |
| 40 | Asb13 | ankyrin repeat and SOCS box-containing 13 | -4.07 | NM_001108420 |
| 41 | RGD1564450 | uncharacterized protein LOC294291 | -4.05 | NM_001106377 |
| 42 | Agpat3 | 1-acyl-sn-glycerol-3-phosphate acyltransferase gamma | -4.04 | NM_001106378 |
| 43 | Bcar3 | breast cancer anti-estrogen resistance protein 3 | -4.03 | NM_001107722 |
| 44 | Ppp1r7 | protein phosphatase 1 regulatory subunit 7 | -4.01 | NM_001009825 |
| 45 | Ghitm | growth hormone-inducible transmembrane protein | -4 | NM_001005908 |
| 46 | Adam23 | disintegrin and metalloproteinase domain-containing protein 23 | -3.99 | NM_001029899 |
| 47 | Ncan | neurocan core protein precursor | -3.94 | NM_031653 |
| 48 | Mamdc4 | apical endosomal glycoprotein precursor | -3.92 | NM_145768 |
| 49 | Lbp | lipopolysaccharide-binding protein precursor | -3.92 | NM_017208 |
| 50 | Tk2 | thymidine kinase 2, mitochondrial | -3.91 | NM_001106166 |

**Supplemental Table 3 Genes with increased methylation that involved into the cardiac muscle cell development by GO analysis**

| **Symbol** | **Gene name** | **log_2_ Fold Change** | **Accession Number** |
| --- | --- | --- | --- |
| Acadm | medium-chain specific acyl-CoA dehydrogenase, mitochondrial precursor | 3.8 | NM_016986 |
| Bmp4 | bone morphogenetic protein 4 precursor | 3.11 | NM_012827 |
| Tbx3 | T-box transcription factor TBX3 | 2.9 | NM_181638 |
| Nkx2-6 | homeobox protein Nkx-2.6 | 1.82 | NM_001127653 |

**Supplemental Table 4 Genes with decreased methylation that involved into the cardiac muscle cell development by GO analysis**

| **Symbol** | **Gene name** | **log_2_ Fold Change** | **Accession Number** |
| --- | --- | --- | --- |
| Sik1 | serine/threonine-protein kinase SIK1 | -4.43 | NM_021693 |
| Rom1 | rod outer segment membrane protein 1 | -4.41 | NM_001009690 |
| Adam15 | disintegrin and metalloproteinase domain-containing protein 15 precursor | -4.35 | NM_020308 |
| Srf | serum response factor | -3.17 | NM_001109302 |
| Pdgfa | platelet-derived growth factor subunit A precursor | -3.11 | NM_012801 |
| Tgfbi | transforming growth factor-beta-induced protein ig-h3 precursor | -3.08 | NM_053802 |
| Elk3 | ETS domain-containing protein Elk-3 | -3.04 | NM_001108743 |
| Camk2d | calcium/calmodulin-dependent protein kinase type II subunit delta | -3.04 | NM_012519 |
| Robo4 | roundabout homolog 4 precursor | -2.98 | NM_181375 |
| S1pr1 | sphingosine 1-phosphate receptor 1 | -2.79 | NM_017301 |
| Atm | serine-protein kinase ATM | -2.5 | NM_001106821 |
| Vdr | vitamin D3 receptor | -2.47 | NM_017058 |
| Sp2 | transcription factor Sp2 | -2.44 | NM_001107045 |
| Hyal1 | hyaluronidase-1 precursor | -2.23 | NM_207616 |
| Ndufv2 | NADH dehydrogenase [ubiquinone] flavoprotein 2, mitochondrial precursor | -2.13 | NM_031064 |
| Popdc2 | popeye domain-containing protein 2 | -2.07 | NM_199113 |
| Adm2 | ADM2 precursor | -1.99 | NM_201426 |
| Ccdc40 | coiled-coil domain-containing protein 40 | -1.97 | NM_001134688 |
| Cdc42 | cell division control protein 42 homolog precursor | -1.8 | NM_171994 |
| P2rx4 | P2X purinoceptor 4 | -1.39 | NM_031594 |
| Fzd9 | frizzled-9 precursor | -1.24 | NM_153305 |
| Wnt8a | wingless-type MMTV integration site family, member 8A precursor | -1.08 | NM_001106155 |
| Aplnr | apelin receptor | -1.03 | NM_031349 |
